# Nationwide Assessment of Knowledge and Perception in Reinforcing Telemedicine in the Age of COVID-19 Among Medical Students From Pakistan

**DOI:** 10.3389/fpubh.2022.845415

**Published:** 2022-03-31

**Authors:** Sana Kazmi, Farah Yasmin, Sarush Ahmed Siddiqui, Muzhgan Shah, Rabbia Tariq, Hamza Nauman, Usama Saeed, Amir Hassan, Muhammad Sohaib Asghar, Tooba Hussain

**Affiliations:** ^1^Department of Internal Medicine, Dow Medical College, Dow University of Health Sciences, Karachi, Pakistan; ^2^Department of Internal Medicine, Bolan Medical College, Quetta, Pakistan; ^3^Department of Internal Medicine, Lady Reading Hospital, Peshawar, Pakistan; ^4^Department of Internal Medicine, Dow University of Health Sciences, Karachi, Pakistan

**Keywords:** COVID-19, pandemic, telemedicine, telehealth, lockdown

## Abstract

The urgency for telemedicine is felt during the COVID-19 pandemic which has rendered the world shut by enforcing quarantines and lockdowns. Many developing countries including Pakistan have inadequate telehealth care services that limited access to rural and remote areas. A cross-sectional survey was carried out among medical students i.e., both preclinical and clinical enrolled in various medical colleges from all provinces of Pakistan to determine their Knowledge, Attitude and Perception regarding the use of Telemedicine during the COVID-19 Pandemic. A total of 398 respondents were included in this preliminary survey. Knowledgeable scores were calculated, from a maximum obtainable score of 7. The mean knowledge was found to be significantly associated with age, province, and year of study (*p*-value < 0.05). Attitude scores were calculated from a maximum obtainable score of 10. All the independent variables failed to reach a significant (*p* < 0.05) association with the mean attitude of respondents about telemedicine. Perception scores were calculated from a maximum obtainable score of 8. Residents of Khyber Pakhtunkhwa are more likely to know about telemedicine than Balochistan (*p* = 0.022) on univariate regression. We identified, lack of knowledge and training for telemedicine in medical institutes. It is crucial to assess the knowledge of medical students regarding telemedicine to comprehend, and evaluate their attitude as future doctors who can play a significant role in establishing telemedicine services in the health care system.

## Introduction

Telemedicine is a relatively modern and a technology driven field that allows health care professionals to exchange medical information *via* telecommunications devices (1). The urgency for telemedicine is drastically felt during the global pandemic due to the COVID-19 crisis which has rendered the world shut by enforcing quarantines and lockdowns. The suspension of elective cases and closure of medical outpatient's departments (OPD) has led to an increase in the health crisis during the COVID-19 pandemic (2). According to a study in the United States, Telemedicine services showed a drastic increase of 683% (102.4 to 801.6 cases per day), between 2nd March and 14th April 2020 (3), thus suggesting an open-minded approach of general population toward implementation of e-health and telehealth services during the COVID-19 pandemic. With a shortage of doctors and healthcare staff and an increased workload in hospitals, the demand for telehealth services has increased as patients are looking for alternate methods like telemedicine in their health care systems.

Studies in developed countries like the United States have shown that use of telehealth can improve quality and effectiveness of health care at a reduced cost and in much less time (4). In rural areas, where there are comparatively few doctors available, telemedicine can improve access to healthcare through reducing the need for patients or doctors to travel (5), which makes telemedicine a possible solution to medical problems in underdeveloped countries. However, valid concerns regarding this technology such as privacy, security risks and misuse of information still exist and pose a serious threat to its credibility (5). Many developing countries including Pakistan have inadequate telehealth care services and poor telecommunication services that limited the access to rural and remote areas. However, the introduction of broadband internet services in remote areas (6), and establishment of telemedicine start-ups such as OlaDoc, find my doctor, Sehat Kahani etc. are raising hope for a promising future of telemedicine services in Pakistan (7).

A study was conducted among doctors in Karachi in order to assess their knowledge and attitude regarding telemedicine. It was found that the understanding of applications of telemedicine by the doctors in Pakistan was average and a large number (98.2%) complained of having no conferences or meetings at their workplace regarding the system, which resulted in insufficient awareness regarding telemedicine guidelines (8). This proves the necessity of raising awareness about this technology and educating the health care professionals so it can be put to the best possible use. It is also noticed that there is a scarcity of literature on telemedicine in Pakistan and to establish a telehealth care system, there is a significant need for conducting more studies, especially among medical students who will be the healthcare professionals of tomorrow. It is therefore crucial to assess the knowledge of medical students regarding telemedicine to comprehend, and evaluate their attitude as future doctors who can play a significant role in establishing telemedicine services in the health care system. Hence, this cross-sectional study aims to assess the knowledge and perception of medical students in different provinces of Pakistan, regarding telemedicine as a method of providing health care facility.

## Methodology

A cross-sectional survey was carried out among medical students i.e., both preclinical and clinical enrolled in various public and private sector medical colleges from all provinces of Pakistan to determine their Knowledge, Attitude and Perception regarding the use of Telemedicine during the COVID-19 Pandemic. The data was collected over a period of 3 months between July 2021 and October 2021. The sample for this study was calculated by using a single population proportion formula using Open-Source Epidemiologic Statistics for Public Health (OpenEpi) version 3, and sample size was calculated to be 384 with a confidence interval (CI) of 95%, and a 5% margin of error. We further added a 10% contingency to the sample size. Thus, a total of 423 responses were collected, out of which 398 were chosen, and 25 forms were rejected due to incomplete submissions.

A self-designed questionnaire was developed after extensive literature review of previous related studies. All efforts were made to make it brief and participant friendly. A total of 25 questions were included. The questionnaire included 4 sections. The first section of the questionnaire consisted of demographic details such as age, gender, name of province, name of medical college, and year of study. The second section included questions about knowledge of telemedicine technology. A Likert scale of 1 to 5 was used to assess the attitude and perception (1 = Strongly disagree 2 = Disagree 3 = Neither agree or disagree 4 = Agree 5 = Strongly Agree) toward telehealth, in the next 2 sections of the questionnaire respectively. The questionnaire was non-validated, and adopted by reviewing various studies conducted on knowledge, attitude and perception of medical students (9, 10).

Participants were recruited using a convenience sampling method. Medical students from all four provinces i.e., Balochistan, Sindh, Khyber Pakhtunkhwa and Punjab were targeted and included in the study. Medical students from Azad Jammu and Kashmir (disputed region between Pakistan, China and India), specialists, surgeons, residents, house officers, dentists, nurses, and those who did not give consent were excluded from the study. A self-administered questionnaire, along with a consent form, was made using google forms and was propagated *via* social media platforms. Due to the spread of the COVID-19 pandemic and the lockdown policy enforced in the country, a physical and paper-based questionnaire was not feasible. Thus, respondents were reached *via* emails and social media platforms such as WhatsApp and Facebook messenger. The credibility of social media was ensured to reduce the bias of receiving irrelevant responses.

Data was analyzed utilizing the Statistical Package for the Social Sciences (SPSS) software, v.20. To summarize the obtained data, the demographic characteristics of respondents were subjected to descriptive statistics (frequency and mean). To assess knowledge, attitude, and perception levels of respondents, a numeric scoring pattern was used (Table 1), and outcome (dependent) variables–knowledge, attitude, and perception–were recorded. Chi-square test was used to test for association between independent variables (demographics) and outcome variables (knowledge, attitude, and perception) at a 95% confidence interval with significant variables (*p* < 0.05) subjected to a logistic regression model. Categorical variables were reported as frequencies with percentages. Means with standard deviation were presented for continuous variables such as age, gender, province, year of study.

**Table 1:** Numeric scoring pattern.

	**Maximum obtainable score**	**Poor**	**Satisfactory**	**Good**
Knowledge	7	0–2	3–4	5–7
Attitude	10	0–3	4–6	7–10
Perception	8	0–2	3–5	6–8

## Results

### Respondent Demographics

A total of 398 respondents were included in this preliminary survey. Out of the total sample *n* = 173 (43.5%) of the sample was male and *n* = 225 (56.5%) were female. Majority of the respondents belonged to the age group of 19–22 years (62.1%) followed by 23-26 years of age group (34.4%) and respondents belonging to age group of 15–18 and 27–30 years were 3.3 and 0.3% respectively. The distribution of the sub samples showed that the clinical respondents (3rd, 4th, and 5th years) formed the majority of the sample (63.6%). Participants of the study mostly belonged to Balochistan (*n* = 105, 26.4%) followed by Sindh (25.1%), Punjab (24.6%) and KPK (23.9%), as shown in Table 2.

**Table 2:** Demographics of sample showing age, gender, province and year of study.

**Demographic**	**Frequency (n)**	**Percentage (%)**
**Age group of respondents**		
15–18-year-old	13	3.3
19–22-year-old	247	62.1
23–26-year-old	137	34.4
27–30-year-old	1	0.3
**Gender of respondents**		
Male	173	43.5
Female	225	56.5
**Province**		
Sindh	100	25.1
Punjab	98	24.6
Balochistan	105	26.4
Khyber Pakhtunkhwa (KPK)	95	23.9
**Year of study**		
1st year	65	16.3
2nd year	80	20.1
3rd year	72	18.1
4th year	76	19.1
5th year	105	26.4

### Knowledge

Respective knowledgeable scores were calculated, from a maximum obtainable score of 7 (Table 1). Most respondents (59%, *n* = 235) had a Good Knowledge about telemedicine (Supplementary Table 4) and social media was the main source of information for most (*n* = 271, 68.1%) responders (Supplementary Table 1). A total of *n* = 193 (48.5%) of the sample appropriately defined Telemedicine as “Distribution of health-related services and information *via* electronic information and telecommunication technologies” and *n* = 150 (37.7%) defined telemedicine as “Practice of caring for patients remotely when the provider and patient are not physically present with each other.” A total of 75.1% (*n* = 299) of the respondents believed that “All specialties can be practiced through telemedicine” and 51.1% believed that “there are no ethical limitation or ruling regarding telemedicine in Pakistan.” Most of the participants (73.9%) of the sample were aware about the fact that telemedicine is being practiced in Pakistan, however, a majority (56.3%) was not aware about hospitals offering proper telemedicine services (Supplementary Table 1). The mean of the Knowledge was compared with respect to the demographical variables (age, gender, province and year of study). The mean knowledge was found to be significantly associated with age, province and year of study (*p* value <0.05) as shown in Supplementary Table 7.

### Attitude

Attitude scores were calculated from a maximum obtainable score of 10 (Table 1). Most respondents (47%, *n* = 187) had a satisfactory Attitude toward telemedicine (Supplementary Table 5) and a majority (*n* = 230, 57.8%) of the respondents were of the opinion that telemedicine services can be effectively provided using video call, voice call and emails (Supplementary Table 2). Out of the 398 responders, 334 (83.9%) were of the opinion (strongly agree and agree) that tertiary care hospitals should have a proper system of telemedicine and 360 (90.5%) strongly agreed or agreed that telemedicine services should be maintained. A total of 146 (36.7%) and 193 (48.5%) strongly agreed, and agreed respectively, that telemedicine can play an essential role in improving the health care system and 73.9% (*n* = 284) believed (agreed or strongly agreed) that they will be making use of the telemedicine system as a future health care worker. Responses for various questions regarding the ethical issues associated with Telemedicine were recorded and it was observed that the majority of respondents (*n* = 347, 87.1%) either agreed or strongly agreed that there should be development of separate ethical laws regarding telemedicine (Supplementary Table 2). All of the independent variables (age, gender, province, background, and year of study) failed to reach significant (*p* < 0.05) association with the mean attitude of respondents about telemedicine, as shown in Supplementary Table 8.

### Perceptions

Perception scores were calculated from a maximum obtainable score of 8 (Table 1). Most respondents (56%, *n* = 223) had a satisfactory attitude toward telemedicine (Supplementary Table 6) with a vast majority (*n* = 391, 98.8%) of the respondents agreeing to the fact that telemedicine can be a helpful tool in the healthcare during the COVID-19 pandemic (Supplementary Table 3). Majority were of the opinion that telemedicine is a cost-effective system that can reduce the burden on tertiary care hospitals and also play a role in managing emergency conditions. Most of the responders (*n* = 269, 67.6%) believed (Strongly agreed or agreed) that Telemedicine is the future of healthcare in Pakistan, however majority were of the opinion that the chances of medical errors will be high in providing consultation *via* telemedicine and it will be a challenge to establish the facility of telemedicine in rural areas of Pakistan (Supplementary Table 3). All of the independent variables (age, gender, province, background, and year of study) were compared with the mean attitude of respondents about telemedicine, and they failed to reach a significant (*p* < 0.05) association, as shown in Supplementary Table 9.

On the Univariate regression model, individuals with age less than or equal to 21 were less likely to know about telemedicine (*p* < 0.001) as shown in Table 3. Similarly, 1st (*p* = 0.001) and 2nd (*p* = 0.011) year medical students were more likely to not know about telemedicine. Those individuals who didn't know the correct definition of telemedicine (*p* < 0.001), and those whose source of information is friends or family (*p* < 0.001) rather than their medical school or media are less likely to know about telemedicine. On the contrary, residents of Khyber Pakhtunkhwa are more likely to know about telemedicine than Balochistan (*p* = 0.022). When the model was adjusted for age and gender, factors found to be associated with increased knowledge are: Knowledge about telemedicine being practiced in Pakistan (*p* < 0.001), Knowledge about any organization or hospital offering proper Telemedicine services in Pakistan (*p* < 0.001), and Knowledge about any ethical limitation or ruling for Telemedicine in Pakistan (*p* = 0.016).

**Table 3:** Regression analysis of characteristics associated with telemedicine knowledge (*n* = 398).

**Study variables**	**Univariate analysis**		***P*-value**	**Multivariate analysis^∧^**		***P*-value**
	**OR**	**95% CI**		**aOR**	**95% CI**	
Gender	
Females	0.772	0.459–1.299	0.330	–	–	–
Males	Ref	–	–	–	–	–
Age	
≤ 21 years	0.295	0.171–0.510	<0.001^*^	–	–	–
> 21 years	Ref	–	–	–	–	–
Region	
Sindh	1.476	0.760–2.867	0.251	1.991	0.980–4.045	0.057
Punjab	1.915	0.949–3.868	0.070	1.552	0.727–3.315	0.256
Khyber Pakhtunkhwa	2.394	1.134–5.053	0.022^*^	1.329	0.575–3.071	0.506
Balochistan	Ref	–	–			
Year of Medical college	
1st year	0.258	0.119–0.558	0.001^*^	0.467	0.145–1.512	0.204
2nd year	0.373	0.174–0.797	0.011^*^	0.631	0.211–1.889	0.411
3rd year	0.876	0.362–2.123	0.770	1.187	0.430–3.277	0.741
4th year	2.007	0.684–5.890	0.205	2.195	0.734–6.564	0.160
5th year	Ref	–	–	Ref	–	–
Response to most appropriate definition of Telemedicine	
Practice of caring for patients remotely when the provider and patient are not physically present with each other	Ref	–	–	Ref	–	–
Distribution of health-related services and information *via* electronic information and telecommunication technologies	0.869	0.415–1.822	0.710	0.754	0.355–1.602	0.463
Searching about disease on internet/Buying medicines online	0.032	0.014–0.074	<0.001	0.033	0.014–0.077	<0.001^*^
Source of information regarding telemedicine	
Medical School	Ref	–	–	Ref	–	–
Social Media/Television	0.949	0.315–2.854	0.925	0.887	0.286–2.745	0.835
Friends/Family	0.127	0.041–0.391	<0.001^*^	0.121	0.065–0.223	<0.001^*^
Knowledge about telemedicine being practiced in Pakistan	
No	Ref	–	–	Ref	–	–
Yes	4.588	2.689–7.828	<0.001^*^	4.365	2.515–7.576	<0.001^*^
Knowledge about which specialties can be practiced with Telemedicine	
Cardiology and psychiatry	1.690	0.489–5.844	0.407	1.919	0.542–6.797	<0.001^*^
Hematology/Oncology, Nephrology, OB/GYN	0.784	0.277–2.214	0.645	0.837	0.286–2.446	0.745
Only psychiatry	1.101	0.507–2.390	0.808	1.297	0.582–2.889	0.524
All the specialties	Ref	–	–	Ref	–	–
Knowledge about any organization or hospital offering proper Telemedicine services in Pakistan	
No	Ref	–	–	Ref	–	–
Yes	6.417	3.181–12.945	<0.001^*^	5.799	2.847–11.808	<0.001^*^
Knowledge about any ethical limitation or ruling for Telemedicine in Pakistan	
No	Ref	–	–	Ref	–	–
Yes	2.055	1.209–3.491	0.008^*^	1.949	1.131–3.357	0.016^*^

* *Indicates significant p-values of < 0.05*.

∧ *Model was adjusted for age and gender*.

*Dependent variable is knowing about telemedicine been answered as “Yes.”*

*OR, odds ratio; aOR, adjusted odds ratio; 95% CI, 95% confidence interval; OB/GYN, obstetrics/gynecology*.

## Discussion

The need of telemedicine and its importance has become clear more than ever during these testing times of pandemic and its adaptation in Pakistan is mostly suboptimal, this study is the first study to assess the knowledge, perception and attitudes regarding telemedicine in medical students across the country to identify any challenge to encounter it for better delivery and understanding of telemedicine services in future.

The results of our study indicate that only 3/5th of our respondents had good knowledge about telemedicine and almost half of them even failed to define it correctly. The suboptimal knowledge of medical students regarding telemedicine has been corroborated by previous studies as well. A cross sectional survey conducted among students of an Austrian medical university showed that 48% of the sample had low to very low information about the subject (11). Another study conducted in China demonstrated that only 41% of medical students had heard of the concept (12). Moreover, our study further highlights that student who are familiar with the concept of telemedicine have a very superficial understanding of its different aspects. While most of the participants recognized that telemedicine is being practiced in Pakistan, more than half of them were not aware of any ethical limitations issued by the government and neither were they familiar with the hospitals or organizations offering telemedicine services. Similar results were observed in France, where 85.6% of the medical students were unaware of the telemedicine regulations (13), as well as in the United States where the complex legislative landscape led to poor comprehension of telemedicine regulations (14).

In Pakistan, this dearth of knowledge may be attributed to the lack of concrete telemedicine regulations, insufficient information dissemination and inadequate marketing of the platforms facilitating this practice. The 1970 Code of Ethics issued by The Pakistan Medical and Dental Council (PMDC) vaguely mentions telemedicine, providing no clarity on its scope and limitations and therefore, failing to establish a solid infrastructure that can familiarize the medical community with the latest developments in this field (15). Therefore, the establishment of a legal safety net is imperative to endorse and authenticate information regarding telemedicine services. In addition to that, consistent efforts need to be channelized by healthcare platforms in effectively publicizing telemedicine initiatives by making use of social media broadcasting and also by notifying patients through email and phone calls.

Another major reason for this unsatisfactory level of knowledge is the absence of telemedicine training courses in the curricula of medical universities, as merely 9.5% of the respondents of our study cited their medical university as the source of knowledge for telemedicine. This is in conjunction with another study in which university lectures were quoted by only 21% of the students as the primary source of information about telemedicine (16). Previous literature suggests that the introduction of telemedicine education and training modules at a German medical university helped students improve their knowledge, skills and attitudes toward telemedicine (17). Thus, it can be established that incorporating such modules into undergraduate medical education can help future doctors get acquainted with the intricacies of the concept. Telemedicine training can be gradually integrated into existing curricular structures such as clinical rotations, lectures, electives, and research opportunities, thereby minimizing any significant additional burden on students (18).

Satisfactory attitudes toward telemedicine were observed in this study. More than 4/5th of the respondents agreed or strongly agreed that telemedicine can improve the healthcare system, and that telemedicine services should be maintained with proper channels in tertiary care hospitals. These results are consistent with the findings of another cross sectional study which demonstrated that 79% of medical students in Austria highly rated the future importance of telemedicine (16). Similarly, a study by Yaghobian et al. showed that 60% of medical students in France recognized the relevance of telemedicine in improving patient care while 82.5% of them also believed that telemedicine may result in improved access to healthcare (13). In our study, 73.9% of the respondents also showed an inclination toward using telemedicine system as a future health care worker. However, this percentage is considerably higher when compared with the surveys conducted prior to the COVID-19 pandemic. In pre-COVID surveys, the percentage of medical students planning to utilize telemedicine in the future amounted to merely 17% in the United States (19) and 36% in France (13). Interestingly, a survey conducted in Australia also demonstrated that despite being enrolled in an e-health course, students had a poor understanding of the relevance of e-health in their future practices (20). This difference between pre-COVID surveys and our study may be attributed to the change in the healthcare landscape during COVID-19 pandemic which resulted in widespread establishment of telemedicine centers, promotion of telemedicine by several healthcare organizations across the world, as well as large scale incorporation of this practice in the government sector for the first time. These factors may have compelled the students to view telemedicine as an essential adjunct strategy for healthcare delivery in the future.

A large percentage of responders in our study believed that accurate diagnoses cannot be made *via* telemedicine. However, recent studies report that diagnostic accuracy of telemedicine is almost equivalent to conventional face to face practice (21, 22). Another important finding of our study is that a vast majority of respondents agreed on having a separate set of ethical laws for the practice of telemedicine. Our study also indicated that most of the students feel that the doctors who practice telemedicine should be paid by the government. These findings are in line with a qualitative study conducted in Netherlands in which students expressed that it is the role of the government to manage reimbursements for eHealth interventions and also to establish a national quality mark to uphold the standard of eHealth services (23). Previous literature also lays emphasis on maintaining established ethical principles when using new forms of communication and technology in healthcare (24). As per a telemedicine survey conducted by the World Health Organization in 2016, Pakistan has no telemedicine laws in place (25). However, Pakistan's National Health Vision 2016–2025 has recognized the key gaps in the health information system and envisioned to channelize more investment into innovative health technologies, such as telemedicine, in an attempt to make healthcare more accessible to everyone (25). Development of concrete guidelines and ethical laws should be the first step in achieving this goal. Moreover, the government needs to overcome the challenges of procuring trained personnel, ensuring licensure of physicians and establishing a secure monitoring system in order to make substantial progress in the field of telemedicine (26).

Multiple studies from across the globe have found varying perceptions of medical personnel on telemedicine. A study in nursing students in Poland found that majority of students perceived that telemedicine improves accessibility to healthcare (27). This is especially true for a developing country such as Pakistan where rural areas often do not have even the basic health facilities available. With the rising use of mobile phones and increasing internet connectivity (~92% access) (28), telemedicine has immense potential for Pakistan. Similar findings were reported from West Bengal by Drs Dey and Bhattacharya whose study reported 76% willingness by post-graduate students to use telemedicine and a 68.7% perception that telemedicine improves access to healthcare services (29). Approximately 64% medical professionals from Ethiopia were found to have a good attitude with regards to telemedicine (4), a result similar to that reported by our study. The general view amongst medical students in Vienna (11), West Bengal (29), and China (12), was that telemedicine reduces the healthcare costs, similar to findings reported in our study. With the advent of telemedicine in a country such as Pakistan, this has enormous implications especially in the post-COVID-19 era where poverty rates have risen, and hence the satisfactory attitude of 56% medical students in our study regarding telemedicine shows hope regarding improved and cheaper access to healthcare. With introduction of mandatory telemedicine courses in the curriculum, perceptions and attitudes can undergo further improvement, as demonstrated by a study in Australia where a statistically significant difference was found in pre and post telemedicine workshop perceptions in students (20).

However, studies have also brought forth some limitations with regards to telemedicine 75.17%, of students in a study conducted by Chen et al. reported the lack of accuracy in data obtained from patients (12), leading to incorrect diagnoses and mismanagement, a finding similar to that obtained by our study. Up to 54% of professionals however felt that telemedicine improves clinical decisions and management (4). In a low-income country like Pakistan, with a low literacy rate (30), communication barriers and technical problems may prevent telemedicine from achieving its full output, a finding similar to what was obtained from postgraduate students in West Bengal (29), and from medical professionals in Ethiopia (4).

### Logistic Regression

With a univariate model of logistic regression, students aged 21 years or younger were less likely to have knowledge about telemedicine (*p* < 0.001). This correlates with the finding of students in their first and second year of medical school being less likely to have adequate knowledge on telemedicine (*p* = 0.001 and *p* = 0.011, respectively). Students from medical colleges in Khyber Pakhtunkhwa province are more likely to have significant knowledge regarding telemedicine as compared to those from Balochistan (*p* = 0.022), which may be attributed to lack of internet services in Balochistan and the literacy rate in Khyber Pakhtunkhwa being 55% as compared to Balochistan's 40% in June 2020 (8). A student who obtained his knowledge on telemedicine from friends and family was statistically less likely to have adequate knowledge as compared to those whose source of information was medical school (*p* < 0.001). Good knowledge is also statistically significant when students were aware about practice of telemedicine within Pakistan (*p* < 0.001), institutes and hospitals where telemedicine is currently practiced (*p* < 0.001) and ethical guidelines for practicing telemedicine within Pakistan (*p* = 0.008), as compared to those who did not have an idea of the above-mentioned parameters. This can result from a greater exposure and awareness about the specialty, hence leading to availability of accurate information.

With age and gender adjusted for in the multivariate analysis, those who defined telemedicine wrongly as ‘searching about diseases on internet/buying medicines online' were more likely to have less knowledge about telemedicine (*p* < 0.001) when compared to those who identified it correctly, as were those who gained their knowledge through friends and family (*p* < 0.001). Statistically medical students with good knowledge were aware about telemedicine being practiced in Pakistan (*p* < 0.001), particularly knowledge about institutions where telemedicine is being practiced within the country (*p* < 0.001) and knowledge of the ethical implications of practicing telemedicine in the country (*p* = 0.016). These differences are attributable to the greater exposure, and an authentic source of information. Students were also more likely to perceive cardiology and psychiatry as specialties more likely to be practiced with telemedicine as compared to others (*p* < 0.001).

## Conclusion

In conclusion, we identified, lack of adequate knowledge and training for telemedicine in medical institutes. Training for telemedicine needs to be added in the curriculum. Moreover, the government and PMDC needs to be proactive and make an elaborate set of laws and regulations for the practice of telemedicine in the country to ensure safety of both physicians and patients.

Unprecedented circumstances due to the pandemic have highlighted the role and need of telemedicine in future too. It is about time telemedicine is integrated in mainstream healthcare provisional services in Pakistan. It is incredibly imperative that medical students are made familiar with the system and are trained adequately for it. Recognizing its limitations for rural areas and a policy to overcome challenges in provision of telemedicine services needs to be implemented.

### Limitations

This cross-sectional survey was conducted using a validated, self-administered electronic questionnaire that was distributed to the medical students of Pakistan *via* different online platforms, and therefore has certain limitations. Firstly, the lack of Internet availability and access to social media platforms may have had an impact on the survey population due to which a bias in reporting results should be considered. The sample was drawn on the basis of convenience and not randomly, it is not a true representative of all medical student population in Pakistan and hence extrapolation is not prudent. Since no pilot study was employed, validity and internal consistency was not checked and Principal Component Analysis (PCA) was also not done.

Nevertheless, the major findings of this study can be used in planning to educate about telemedicine platforms. The study identifies concerns among the participants regarding use of telemedicine, and hence provides a valuable important perspective for possible interventional educational programs to enhance incorporation of telemedicine in the healthcare sector. This study also serves an important baseline to conduct further studies in a larger population to obtain an insight on the behavior, and public perception of Telemedicine. Lastly to the best of our knowledge, this is the first study conducted to determine the knowledge and perception toward telemedicine among medical students from all four provinces of Pakistan. Overall, the study is unique and not much literature is available but has limited applicability for the general audience. Hopefully, this will lead to the development of policies and guidelines for telemedicine in Pakistan.

## Data Availability Statement

The raw data supporting the conclusions of this article will be made available by the authors, without undue reservation.

## Ethics Statement

Ethical review and approval was not required for the study on human participants in accordance with the local legislation and institutional requirements. The patients/participants provided their written informed consent to participate in this study.

## Author Contributions

SK, FY, SS, MS, and RT: conception of the study, major drafting of the work, and final approval and agreeing to the accuracy of the work. HN, US, and AH: drafting of the work, data collection, final approval, and agreeing to the accuracy of the work. MA and TH: drafting of the work, reviewing, editing, and final approval and agreeing to the accuracy of the work. All authors have read and approved the final version of the manuscript.

## Conflict of Interest

The authors declare that the research was conducted in the absence of any commercial or financial relationships that could be construed as a potential conflict of interest.

## Publisher's Note

All claims expressed in this article are solely those of the authors and do not necessarily represent those of their affiliated organizations, or those of the publisher, the editors and the reviewers. Any product that may be evaluated in this article, or claim that may be made by its manufacturer, is not guaranteed or endorsed by the publisher.
