# Supplementary material for: Nationwide Assessment of Knowledge and Perception in Reinforcing Telemedicine in the Age of COVID-19 Among Medical Students From Pakistan
Source: Front Public Health. 2022 Mar 31;10:845415. doi: 10.3389/fpubh.2022.845415 (PMC9008574; doi:10.3389/fpubh.2022.845415)
Supplement: Supplementary file 1 [file Data_Sheet_1.docx]

**Supplementary Table 1: Knowledge of Medical students of Pakistan regarding Telemedicine.**

| **Sr No.** | **Knowledge Questionnaire** | | **N(%)** |
| --- | --- | --- | --- |
| 1. | Do you know what is Telemedicine? | Yes | 325(81.7) |
|  |  | No | 73(18.3) |
| 2. | If yes, what is its most appropriate definition? | Searching about disease on internet | 20(5.0) |
|  |  | Buying medicines online | 1(0.3) |
|  |  | Practice of caring for patients remotely when the provider and patient are not physically present with each other | 150(37.7) |
|  |  | Distribution of health-related services and information via electronic information and telecommunication technologies | 193(48.5) |
| 3. | From what source did you hear about Telemedicine? | Social media | 271(68.1) |
|  |  | Your Medical school | 38(9.5) |
|  |  | Television | 10(2.5) |
|  |  | From a friend | 79(19.8) |
| 4. | Is Telemedicine being practiced in Pakistan? | Yes | 294(73.9) |
|  |  | No | 104(26.1) |
| 5. | Do you know any organization or hospital offering proper Telemedicine services in Pakistan? | Yes | 174(43.7) |
|  |  | No | 224(56.3) |
| 6. | What specialties can be practiced with Telemedicine | All the specialties | 299(75.1) |
|  |  | Only psychiatry | 52(13.1) |
|  |  | Cardiology and psychiatry | 25(6.3) |
|  |  | Hematology/Oncology, Nephrology, OB/GYN | 22(5.5) |
| 7. | Is there any ethical limitation or ruling for Telemedicine in Pakistan? | Yes | 193(48.5) |
|  |  | No | 205(51.5) |

**Supplementary Table 2: Attitude of Medical students of Pakistan regarding Telemedicine.**

| **Sr No.** | **Attitude Questionnaire** | | **N (%)** |
| --- | --- | --- | --- |
| 1. | Do you think proper diagnoses can be made without physical examination? | a) Strongly Agree | 8(2.0) |
|  |  | b) Agree | 78(19.6) |
|  |  | c) Neither agree or disagree | 128(32.2) |
|  |  | d) Disagree | 138(34.7) |
|  |  | e) Strongly Disagree | 46(11.6) |
| 2. | Do you think tertiary care hospitals should have proper channel for telemedicine? | a) Strongly Agree | 137(34.4) |
|  |  | b) Agree | 197(49.5) |
|  |  | c) Neither agree or disagree | 49(12.3) |
|  |  | d) Disagree | 13(3.3) |
|  |  | e) Strongly Disagree | 2(0.5) |
| 3. | Do you think doctor-patient interaction should be recorded? | a) Strongly Agree | 106(26.6) |
|  |  | b) Agree | 149(37.4) |
|  |  | c) Neither agree or disagree | 66(16.6) |
|  |  | d) Disagree | 47(11.8) |
|  |  | e) Strongly Disagree | 30(7.5) |
| 4. | Do you think Telemedicine service should be maintained? | a) Strongly Agree | 154(38.7) |
|  |  | b) Agree | 206(51.8) |
|  |  | c) Neither agree or disagree | 30(7.5) |
|  |  | d) Disagree | 7(1.8) |
|  |  | e) Strongly Disagree | 1(0.3) |
| 5. | Do you think it can improve quality of care of health? | a) Strongly Agree | 146(36.7) |
|  |  | b) Agree | 193(48.5) |
|  |  | c) Neither agree or disagree | 51(12.8) |
|  |  | d) Disagree | 6(1.5) |
|  |  | e) Strongly Disagree | 2(0.5) |
| 6. | Do you think separate set of ethical laws and rulings should be made for practice of Telemedicine? | a) Strongly Agree | 157(39.4) |
|  |  | b) Agree | 190(47.7) |
|  |  | c) Neither agree or disagree | 38(9.5) |
|  |  | d) Disagree | 11(2.8) |
|  |  | e) Strongly Disagree | 2(0.5) |
| 7. | Do you think there should be time limit for each doctor-patient interaction? | a) Strongly Agree | 75(18.8) |
|  |  | b) Agree | 138(34.7) |
|  |  | c) Neither agree or disagree | 98(24.6) |
|  |  | d) Disagree | 63(15.8) |
|  |  | e) Strongly Disagree | 24(6.0) |
| 8. | Do you see yourself as future health-care professional using the service of telemedicine? | a) Strongly Agree | 109(27.4) |
|  |  | b) Agree | 185(46.5) |
|  |  | c) Neither agree or disagree | 78(19.6) |
|  |  | d) Disagree | 24(6.0) |
|  |  | e) Strongly Disagree | 2(0.5) |
| 9. | Do you think service of telemedicine should be free and doctors should be paid from Government? | a) Strongly Agree | 163(41.0) |
|  |  | b) Agree | 158(39.7) |
|  |  | c) Neither agree or disagree | 55(13.8) |
|  |  | d) Disagree | 21(5.3) |
|  |  | e) Strongly Disagree | 1(0.3) |
| 10. | How should Telemedicine be practiced? | a) Video Call | 142(35.7) |
|  |  | b) Voice Call | 25(6.3) |
|  |  | c) Emails | 1(0.3) |
|  |  | d) All of above | 230(57.8) |

**Supplementary Table 3: Perception of Medical students of Pakistan regarding Telemedicine.**

| **Sr No.** | **Perception Questionnaire** | | **N (%)** |
| --- | --- | --- | --- |
| 1. | Do you think Telemedicine can be helpful in covid-19 pandemic crises? | a) Strongly Agree | 253(63.6) |
|  |  | b) Agree | 138(34.7) |
|  |  | c) Neither agree or disagree | 7(1.8) |
|  |  | d) Disagree | 0 |
|  |  | e) Strongly Disagree | 0 |
| 2. | Do you Telemedicine services will be cost effective and improve access to health services? | a) Strongly Agree | 152(38.2) |
|  |  | b) Agree | 191(48.0) |
|  |  | c) Neither agree or disagree | 40(10.1) |
|  |  | d) Disagree | 14(3.5) |
|  |  | e) Strongly Disagree | 1(0.3) |
| 3. | Do you think there is relatively more chance of medical error? | a) Strongly Agree | 85(21.4) |
|  |  | b) Agree | 188(47.2) |
|  |  | c) Neither agree or disagree | 102(25.6) |
|  |  | d) Disagree | 21(5.3) |
|  |  | e) Strongly Disagree | 2(0.5) |
| 4. | Do you think proper provision of telemedicine can help reduce burden of tertiary care hospitals? | a) Strongly Agree | 129(32.4) |
|  |  | b) Agree | 203(51.0) |
|  |  | c) Neither agree or disagree | 57(14.3) |
|  |  | d) Disagree | 7(1.8) |
|  |  | e) Strongly Disagree | 2(0.5) |
| 5. | Do you Telemedicine services as future of health care in Pakistan? | a) Strongly Agree | 89(22.4) |
|  |  | b) Agree | 180(45.2) |
|  |  | c) Neither agree or disagree | 92(23.1) |
|  |  | d) Disagree | 34(8.5) |
|  |  | e) Strongly Disagree | 3(0.8) |
| 6. | Do you think there is third party needed to maintain communication network other than doctors? | a) Strongly Agree | 110(27.6) |
|  |  | b) Agree | 166(41.7) |
|  |  | c) Neither agree or disagree | 77(19.3) |
|  |  | d) Disagree | 32(8.0) |
|  |  | e) Strongly Disagree | 13(3.3) |
| 7. | Do you think telemedicine is feasible in rural and remote areas of the country? | a) Strongly Agree | 71(17.8) |
|  |  | b) Agree | 92(23.1) |
|  |  | c) Neither agree or disagree | 96(24.1) |
|  |  | d) Disagree | 110(27.6) |
|  |  | e) Strongly Disagree | 29(7.3) |
| 8. | Do you think medical emergencies can be addressed via Telemedicine? | a) Strongly Agree | 68(17.1) |
|  |  | b) Agree | 104(26.1) |
|  |  | c) Neither agree or disagree | 86(21.6) |
|  |  | d) Disagree | 66(16.6) |
|  |  | e) Strongly Disagree | 74(18.6) |

**Supplementary Table 4: Grading of Knowledge of Medical students of Pakistan regarding Telemedicine**

| **Demographic** | **Poor Knowledge** | **Satisfactory Knowledge** | **Good Knowledge** | **p value** |
| --- | --- | --- | --- | --- |
| **Age group**  15-18 years old  19-22 years old  23-26 years old  27-30 years old | 2 (15.3%)  23 (9.3%)  4 (2.9%)  0 (0%) | 7 (53.8%)  89 (36.0%)  38 (27.7%)  0 (0%) | 4 (30.8%)  135 (54.6%)  95 (69.3%)  1 (%) | **0.018** |
| **Gender**  Male  Female | 11 (6.4%)  18 (8.0%) | 56 (32.3%)  78 (34.7%) | 106 (61.3%)  129 (57.3%) | 0.680 |
| **Province**  Sindh  Punjab  Balochistan  Khyber Pakhtunkhwa | 9 (9.0%)  3 (3.1%)  11 (10.5%)  6 (6.3%) | 36 (36.0%)  24 (24.5%)  45 (42.9%)  29 (30.5%) | 55 (55.0%)  71 (72.4%)  49 (46.7%)  60 (63.2%) | **0.013** |
| **Year of study**  1st year  2nd year  3rd year  4th year  5th year | 12 (18.5%)  8 (10.0%)  4 (5.5%)  2 (2.6%)  3 (2.9%) | 26 (40.0%)  44 (55.0%)  18 (25.0%)  19 (25.0%)  27 (25.7%) | 27 (41.5%)  28 (35.0%)  50 (69.4%%)  55 (72.4%)  75 (71.4%) | **0.001** |
| **Total** | 29 (7.3%) | 134 (33.7%) | **235 (59.0%)** |  |

**Supplementary Table 5: Grading of Attitude of Medical students of Pakistan regarding Telemedicine.**

| **Demographic** | **Poor Attitude** | **Satisfactory Attitude** | **Good Attitude** | **p value** |
| --- | --- | --- | --- | --- |
| **Age group**  15-18 years old  19-22 years old  23-26 years old  27-30 years old | 6 (46.1%)  93 (37.6%)  44 (32.1%)  0 (%) | 6 (46.1%)  116 (47.0%)  64 (46.7%)  1 (100.0%) | 1 (7.7%)  38 (15.4%)  29 (21.2%)  0 (0%) | 0.584 |
| **Gender**  Male  Female | 57 (32.9%)  86 (38.2%) | 85 (49.1%)  102 (45.3%) | 31 (17.9%)  37 (16.4%) | 0.554 |
| **Province**  Sindh  Punjab  Balochistan  Khyber Pakhtunkhwa | 41 (41.0%)  35 (35.7%)  35 (33.3%)  32 (33.7%) | 43 (43.0%)  44 (44.9%)  53 (50.1%)  47 (49.8%) | 16 (16.0%)  19 (19.4%)  17 (16.2%)  16 (16.8%) | 0.890 |
| **Year of study**  1st year  2nd year  3rd year  4th year  5th year | 24 (36.9%)  29 (36.2%)  28 (38.9%)  31 (40.8%)  31 (29.5%) | 32 (49.2%)  37 (46.2%)  29 (40.3%)  36 (47.4%)  53 (50.5%) | 9 (13.8%)  14 (17.5%)  15 (20.8%)  9 (11.8%)  21 (20.0%) | 0.683 |
| **Total** | 143 (35.9%) | **187 (47.0%)** | 68 (17.1%) |  |

**Supplementary Table 6: Grading of Perception of Medical students of Pakistan regarding Telemedicine**

| **Demographic** | **Poor Perception** | **Satisfactory Perception** | **Good Perception** | **p value** |
| --- | --- | --- | --- | --- |
| **Age group**  15-18 years old  19-22 years old  23-26 years old  27-30 years old | 5 (38.5%)  108 (43.7%)  51 (37.2%)  1 (100.0%) | 8 (61.5%)  131 (53.0%)  84 (61.3%)  0 (0%) | 0 (0%)  8 (80.0%)  2 (20.0%)  0 (0%) | 0.552 |
| **Gender**  Male  Female | 69 (39.9%)  96 (42.7%) | 98 (56.6%)  125 (55.5%) | 6 (60.0%)  4 (40.0%) | 0.518 |
| **Province**  Sindh  Punjab  Balochistan  Khyber Pakhtunkhwa | 43 (43.0%)  31 (31.6%)  51 (48.6%)  40 (42.1%) | 53 (53.0%)  66 (67.3%)  52 (49.5%)  52 (54.7%) | 4 (40.0%)  1 (10.0%)  2 (20.0%)  3 (30.0%) | 0.178 |
| **Year of study**  1st year  2nd year  3rd year  4th year  5th year | 30 (46.1%)  33 (41.2%)  34 (47.2%)  28 (36.8%)  40 (38.1%) | 34 (52.3%)  46 (57.5%)  34 (47.2%)  46 (60.5%)  63 (60.0%) | 1 (10.0%)  1 (10.0%)  4 (40.0%)  2 (20.0%)  2 (20.0%) | 0.545 |
| **Total** | 165(41.5%) | **223(56%)** | 10(2.5%) |  |

**Supplementary Table 7: Comparison of mean Knowledge scores of Medical students of Pakistan regarding Telemedicine**

| **Demographic** | **Mean ±SD** | **p value** |
| --- | --- | --- |
| **Age group**  15-18 years old  19-22 years old  23-26 years old  27-30 years old | 4.08±1.441  4.59±1.487  4.99±1.300  7.00±. | **0.012** |
| **Gender**  Male  Female | 4.80±1.459  4.65±1.426 | 0.278 |
| **Province**  Sindh  Punjab  Balochistan  Khyber Pakhtunkhwa | 4.57±1.402  5.03±1.280  4.41±1.591  4.87±1.393 | **0.014** |
| **Year of study**  1st year  2nd year  3rd year  4th year  5th year | 4.11±1.501  4.04±1.326  5.01±1.389  5.37±1.403  4.92±1.207 | **0.001** |

**Supplementary Table 8: Comparison of mean attitude scores of Medical students of Pakistan regarding Telemedicine**

| **Demographic** | **Mean ±SD** | **p value** |
| --- | --- | --- |
| **Age group**  15-18 years old  19-22 years old  23-26 years old  27-30 years old | 3.85±2.193  4.27±2.253  4.49±2.377  4.00±. | 0.564 |
| **Gender**  Male  Female | 4.44±2.111  4.25±2.420 | 0.346 |
| **Province**  Sindh  Punjab  Balochistan  Khyber Pakhtunkhwa | 4.14±2.383  4.38±2.538  4.44±2.188  4.37±2.042 | 0.693 |
| **Year of study**  1st year  2nd year  3rd year  4th year  5th year | 4.38±2.163  4.30±2.184  4.40±2.499  3.96±2.242  4.54±2.341 | 0.595 |

**Supplementary Table 9: Comparison of mean perception scores of Medical students of Pakistan regarding Telemedicine**

| **Demographic** | **Mean ±SD** | **p value** |
| --- | --- | --- |
| **Age group**  15-18 years old  19-22 years old  23-26 years old  27-30 years old | 2.77±1.481  2.88±1.440  2.90±1.431  1.00±. | 0.529 |
| **Gender**  Male  Female | 2.92±1.488  2.84±1.398 | 0.749 |
| **Province**  Sindh  Punjab  Balochistan  Khyber Pakhtunkhwa | 2.95±1.559  3.01±1.388  2.62±1.368  2.95±1.409 | 0.130 |
| **Year of study**  1st year  2nd year  3rd year  4th year  5th year | 2.78±1.463  2.81±1.370  2.92±1.499  2.87±1.417  2.96±1.461 | 0.871 |
